# Projection-defined hypothalamic outputs differentially regulate thermogenesis and lipolysis

**DOI:** 10.1073/pnas.2535878123

**Published:** 2026-05-27

**Authors:** Hyeonyoung Min, Qi Zheng, Yunlei Yang

**Affiliations:** ^a^ Department of Medicine Division of Endocrinology, Albert Einstein College of Medicine, Bronx, NY 10461; ^b^ Department of Neuroscience, Albert Einstein College of Medicine, Bronx, NY 10461; ^c^ Einstein-Mount Sinai Diabetes Research Center, Albert Einstein College of Medicine, Bronx, NY 10461; ^d^ The Fleischer Institute for Diabetes and Metabolism, Albert Einstein College of Medicine, Bronx, NY 10461

**Keywords:** VMH, thermogenesis, lipolysis, PVT, rPAG

## Abstract

The ventromedial hypothalamus (VMH) is a key regulator of energy homeostasis, linking central neural activity to peripheral metabolic function. Within the VMH, neurons expressing steroidogenic factor-1 (SF-1; VMH^SF1^) are essential for regulating energy expenditure, yet the projection-defined pathways through which they differentially control distinct adipose depots remains unclear. Here, we use projection-specific optogenetic and chemogenetic manipulations in SF1-Cre mice to identify two anatomically and functionally distinct VMH^SF1^ output pathways with complementary metabolic roles. Activation of the VMH^SF1^→rostral periaqueductal gray (rPAG) projection selectively stimulates thermogenesis in brown adipose tissue, elevating its temperature and thermogenic gene expression, whereas activation of the VMH^SF1^→paraventricular thalamus (PVT) pathway promotes lipolysis in white adipose tissue without engaging thermogenic programs. Both effects require intact innervation and target-region activity. These findings reveal that VMH^SF1^ neurons direct distinct hypothalamic output pathways to differentially regulate thermogenesis and lipid mobilization, delineating a modular neural framework for flexible, state-dependent coordination of energy expenditure and substrate utilization.

## Significance

Energy homeostasis depends on precise coordination between hypothalamic outputs and peripheral metabolic tissues. The ventromedial hypothalamus (VMH) is central to this regulation, yet the pathways linking it to distinct adipose depots remain unclear. We identify two anatomically segregated VMH^SF1^ projection pathways with dissociable metabolic functions: a VMH^SF1^ →rPAG projection that drives brown fat thermogenesis, and a VMH^SF1^ →PVT pathway that promotes white fat lipolysis. These findings reveal a projection-defined, modular organization of hypothalamic output that partitions thermogenic and lipolytic regulation. This projection-level framework advances our understanding of central metabolic control and highlights pathway-specific targets for restoring energy balance in obesity and related disorders

The maintenance of whole-body energy balance depends on tightly coordinated communication between the central nervous system and peripheral metabolic organs. The hypothalamus receives and integrates hormonal, thermal, and nutrient-related cues, and in turn adjusts autonomic output to control energy expenditure and substrate use (1, 2). Among hypothalamic nuclei, the ventromedial hypothalamus (VMH) plays a central role in metabolic regulation, influencing both brown and white adipose tissues primarily through sympathetic pathways (3, 4). Within this region, steroidogenic factor-1-expressing neurons (SF-1/NR5A1; VMH^SF1^) represent a key population involved in the central control of energy metabolism.

VMH^SF1^ neurons respond to multiple peripheral signals, such as leptin, insulin, and thyroid hormones, to regulate glucose homeostasis, feeding behavior, and thermogenesis (5–7). Activation of VMH^SF1^ neurons increases sympathetic input to interscapular brown adipose tissue (iBAT), which promotes UCP1-mediated thermogenesis and raises energy expenditure (6, 8–13). By contrast, suppressing or ablating these neurons lowers sympathetic activity, diminishes thermogenic responses, and contributes to obesity (14–18). Although substantial evidence supports a role for VMH^SF1^ neurons in controlling energy expenditure, the downstream pathways that enable selective regulation of different adipose depots remain poorly defined.

Anatomical tracing studies have shown that VMH^SF1^ neurons send projections to several diencephalic and midbrain targets, including the periaqueductal gray (PAG) and the paraventricular thalamus (PVT) (7, 19). Traditionally linked to autonomic and defensive functions, the PAG is part of a thermoregulatory circuitry that conveys hypothalamic signals to medullary sympathetic premotor neurons (20–23). In comparison, the PVT acts as an integrative center connecting hypothalamic, limbic, and brainstem networks involved in arousal, stress responses, and metabolic regulation (24–30). These anatomical and functional differences suggest that discrete VMH^SF1^ projection pathways may separately influence thermogenesis and lipid mobilization. Nevertheless, it remains unknown whether VMH^SF1^ output pathways provide projection-specific control over adipose metabolism.

To investigate this, we applied projection-specific optogenetic and chemogenetic strategies to define the roles of the VMH^SF1^→rPAG and VMH^SF1^→PVT pathways in regulating adipose tissue metabolism.

## Results

### Selective Activation of VMH^SF1^→rPAG Projections Enhances Thermogenesis in Brown Adipose Tissue.

**Fig. 1.**
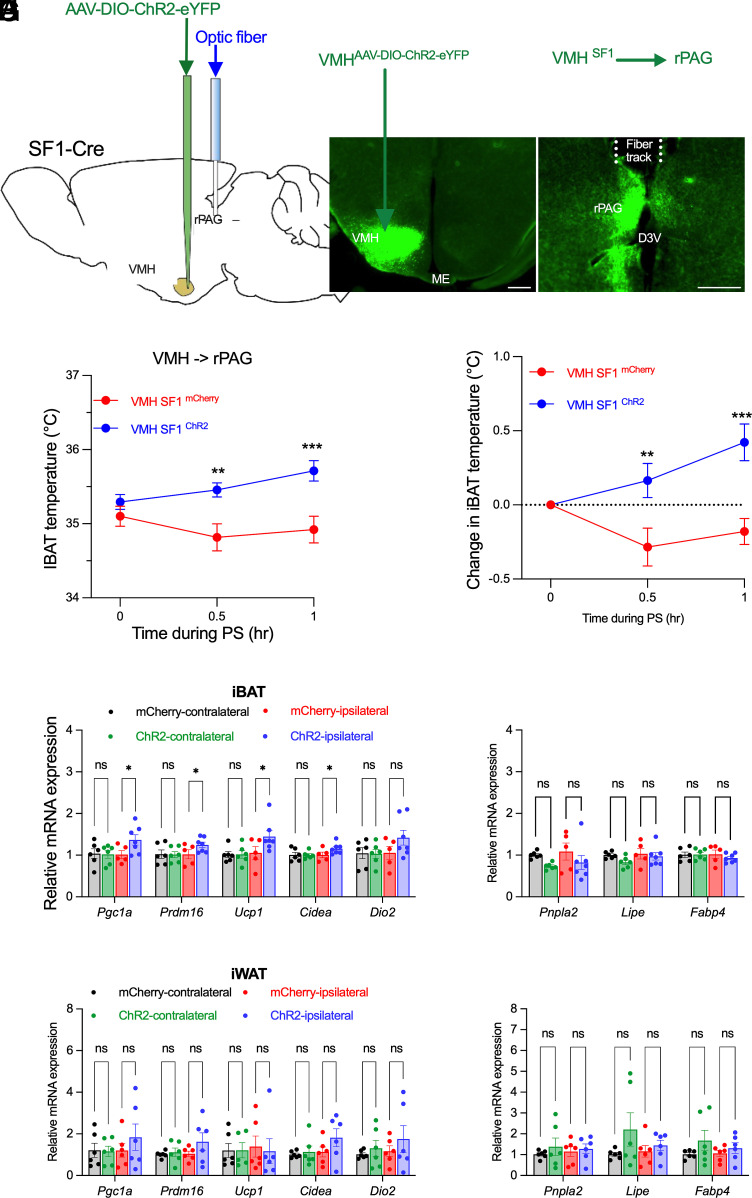
Optogenetic activation of VMH^SF1^→rPAG projections promotes thermogenic responses in brown adipose tissue. (*A*) Schematic of viral delivery into the VMH (AAV-DIO-mCherry or AAV-DIO-ChR2-eYFP) and optic fiber implantation above the rPAG for projection-specific photostimulation. (*B* and *C*) Representative fluorescence images showing ChR2 expression in VMH^SF1^ neurons (*B*) and terminal projections within the rPAG (*C*). (Scale bar, 300 µm.) (*D* and *E*) iBAT temperature (*D*) and temperature change (*E*) before and during photostimulation (0, 0.5, 1 h) in ChR2 (n = 7) versus mCherry (n = 5) mice. (*F* and *G*) quantitative PCR (qPCR) analysis of thermogenic (*Pgc1α*, *Prdm16*, *Ucp1*, *Cidea*, and *Dio2*) (*F*) and lipolytic (*Pnpla2*, *Lipe*, and *Fabp4*) (*G*) gene expression in iBAT from mCherry and ChR2 mice. (*H* and *I*) qPCR analysis of thermogenic (*H*; *Pgc1α*, *Prdm16*, *Ucp1*, *Cidea*, and *Dio2*) and lipolytic (*I*; *Pnpla2*, *Lipe*, and *Fabp4*) gene expression in iWAT from mCherry and ChR2 mice. Data are presented as mean ± SEM. Statistical analysis by two-way ANOVA with Sidak’s post hoc test (*D*–*I*); each dot represents one animal. *P < 0.05, **P < 0.01, ***P < 0.001, n.s., not significant.

To determine whether VMH^SF1^ projections to the rPAG regulate thermogenesis, SF1-Cre mice received stereotaxic injections of Cre-dependent AAV-ChR2-eYFP or control AAV-mCherry into the VMH, followed by implantation of optical fibers above the rPAG (Fig. 1*A*). Robust terminal expression within the rPAG was confirmed by fluorescence microscopy (Fig. 1 *B* and *C*). Projection-specific photostimulation (20 Hz) significantly increased iBAT temperature at 30 and 60 min poststimulation in ChR2-expressing mice (Fig. 1 *D* and *E*). Consistently, mRNA levels of thermogenic genes (*Pgc1α*, *Prdm16*, *Ucp1*, and *Cidea*) were elevated in the ipsilateral iBAT compared with mCherry controls and the contralateral depots (Fig. 1*F*). In contrast, expression of lipolytic genes (*Pnpla2*, *Lipe*, and *Fabp4*) (Fig. 1*F*) and glycerol release (*SI Appendix*, Fig. S1*A*) remained unchanged in iBAT depots, and no significant changes were detected in inguinal white adipose tissue (iWAT) (Fig. 1 *H* and *I*). These findings indicate that activation of the VMH^SF1^→rPAG pathway selectively promotes thermogenesis in brown adipose tissue without affecting white adipose lipid mobilization.

### Activation of VMH^SF1^→PVT Projections Induces Lipolysis in iWAT without Thermogenic Activation in iBAT.

**Fig. 2.**
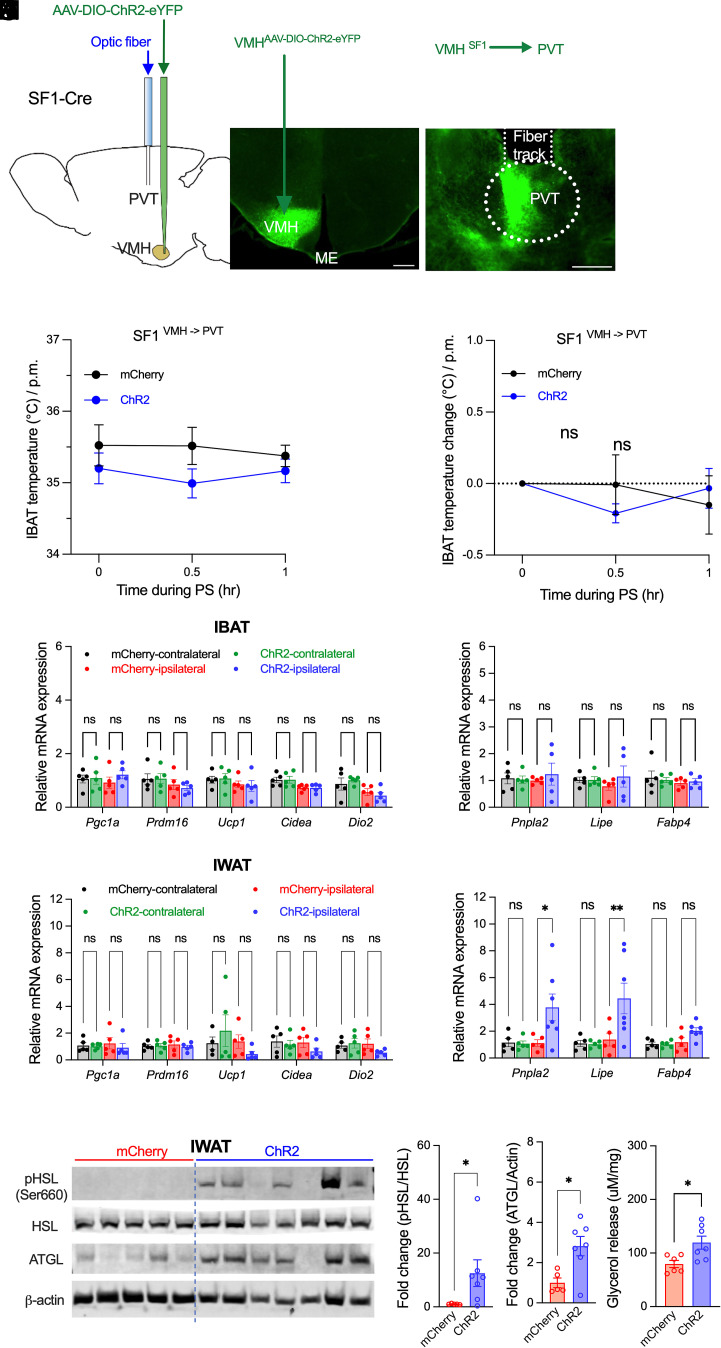
Optogenetic activation of VMH^SF1^→PVT projections promotes lipolysis in white adipose tissue. (*A*) Schematic of viral delivery into the VMH (AAV-DIO-mCherry or AAV-DIO-ChR2-eYFP) and optic fiber implantation above the PVT for projection-specific photostimulation. (*B* and *C*) Representative fluorescence images showing ChR2 expression in VMH^SF1^ neurons (*B*) and terminal projections within the PVT (*C*). (Scale bar, 300 µm.) (*D* and *E*) iBAT temperature (*D*) and temperature change (*E*) before and during photostimulation (0, 0.5, 1 h) in ChR2 vs. mCherry mice (n = 6 each group). (*F* and *G*) qPCR analysis of thermogenic (*Pgc1α*, *Prdm16*, *Ucp1*, *Cidea*, and *Dio2*) (*F*) and lipolytic (*Pnpla2*, *Lipe*, and *Fabp4*) (*G*) gene expression in iBAT from mCherry and ChR2 mice. (*H* and *I*) qPCR analysis of thermogenic (*H*; *Pgc1α*, *Prdm16*, *Ucp1*, *Cidea*, and *Dio2*) and lipolytic (*I*; *Pnpla2*, *Lipe*, and *Fabp4*) gene expression in iWAT from mCherry and ChR2 mice. (*J*–*L*) Representative western blots showing ATGL, HSL, and phosphorylated HSL (p-HSL) protein levels in iWAT from mCherry and ChR2 mice (*J*). Quantification of fold changes in p-HSL relative to total HSL (*K*) and ATGL relative to β-actin (*L*). (*M*) Glycerol release from iWAT (mCherry vs. ChR2). Data are presented as mean ± SEM. Statistical analyses were performed using two-way ANOVA with Sidak’s post hoc test (*D*–*I*) or two-tailed Student’s *t* test (*K*–*M*); each dot represents one animal. *P < 0.05, **P < 0.01, n.s., not significant.

To assess the role of the VMH^SF1^→PVT pathway in adipose metabolism, SF1-Cre mice were injected with the Cre-dependent AAV-ChR2-eYFP into the VMH and implanted with an optic fiber above the PVT (Fig. 2 *A*–*C*). Photostimulation of VMH^SF1^ projections in the PVT did not alter iBAT temperature (Fig. 2 *D* and *E*) or thermogenic gene expression (*Pgc1α*, *Prdm16*, *Ucp1*, *Cidea*; Fig. 2*F*). Similarly, lipolytic gene expression and glycerol release in iBAT were unchanged (Fig. 2*G* and *SI Appendix*, Fig. S2). In contrast, whereas thermogenic gene expression remained unaltered (Fig. 2*H*), photostimulation significantly increased expression of lipolytic genes (*Pnpla2*, *Lipe*, and *Fabp4*) in iWAT (Fig. 2*I*). Western blot analysis confirmed elevated adipose triglyceride lipase (ATGL) and phosphorylated hormone-sensitive lipase (p-HSL) protein levels (Fig. 2 *J*–*L*), and ex vivo assays demonstrated enhanced glycerol release (Fig. 2*M*). Together, these data indicate that VMH^SF1^→PVT activation selectively stimulates lipid mobilization in white adipose tissue without eliciting thermogenic activation in brown fat.

### Chemogenetic Inhibition of rPAG Neurons Abolishes VMH^SF1^→ rPAG-Induced Thermogenesis.

**Fig. 3.**
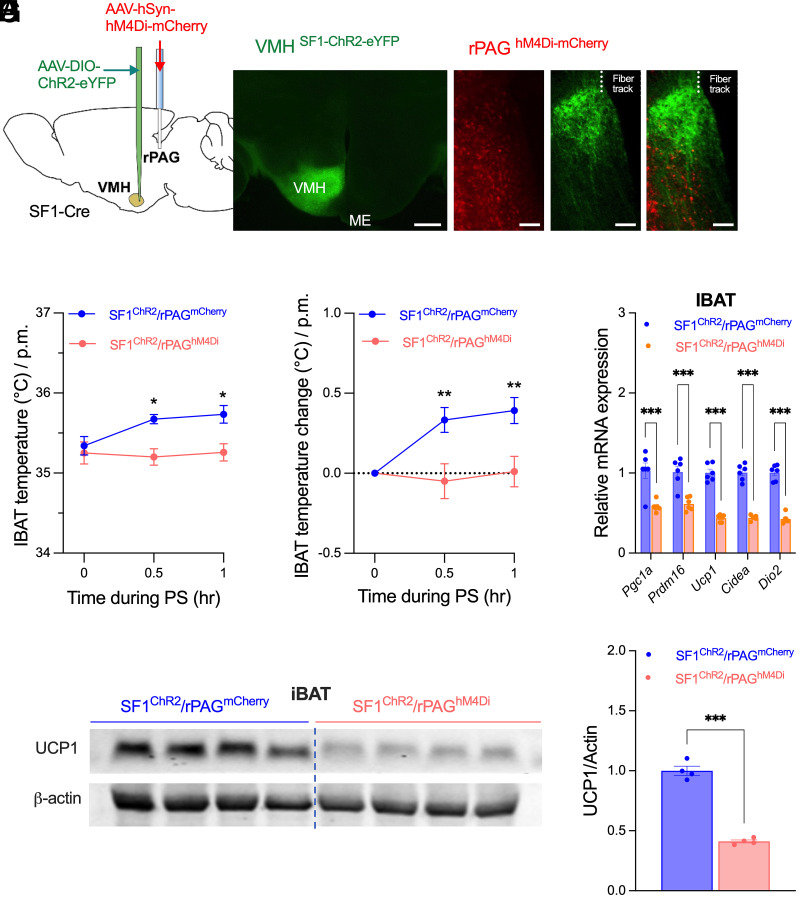
Chemogenetic inhibition of rPAG neurons abolishes VMH^SF1^→rPAG-driven thermogenesis in brown adipose tissue. (*A*) Schematic of viral delivery into the VMH (AAV-DIO-ChR2-eYFP) and rPAG (AAV-hSyn-hM4Di or AAV-hSyn-mCherry), with optic fiber implantation above the rPAG for projection-specific photostimulation. Mice received a single intraperitoneal injection of J60 (1 mg/kg) before the 2-h photostimulation. (*B* and *C*) Representative fluorescence images showing ChR2 expression in VMH^SF1^ neurons (*B*) and terminal projections within the rPAG along with hM4Di expression in rPAG neurons (*C*). [Scale bars, 300 µm (*B*) and 100 µm (*C*).] (*D* and *E*) iBAT temperature (*D*) and temperature change (*E*) before and during photostimulation (0, 0.5, 1 h) in rPAG mCherry (n = 6)- and hM4Di (n = 5)-transduced mice. Mice received a single intraperitoneal injection of J60 (1 mg/kg) 30 min before photostimulation onset. (*F*) qPCR analysis of thermogenic (*Pgc1α*, *Prdm16*, *Ucp1*, *Cidea*, and *Dio2*) gene expression in iBAT. (*G* and *H*) Representative western blots showing UCP1 and actin protein levels in iBAT from rPAG mCherry- and hM4Di-transduced mice (*G*). Quantification of fold changes in UCP1 relative to β-actin (*H*) (n = 4 per group). Data are presented as mean ± SEM. Statistical analyses were performed using two-way ANOVA with Sidak’s post hoc test (*D*–*F*) or two-tailed Student’s *t* test (*H*); each dot represents one animal. *P < 0.05, **P < 0.01, ***P < 0.001.

To determine whether rPAG neuronal activity is required for the VMH^SF1^→rPAG-mediated thermogenesis, AAV-hSyn-hM4Di or control AAV-hSyn-mCherry was injected into the rPAG, and Cre-dependent AAV-ChR2-eYFP was delivered to the VMH (Fig. 3 *A*–*C*). Administration of the DREADD agonist JHU37160 (J60; 1 mg kg^−1^, i.p.) before photostimulation abolished stimulation-induced increases in iBAT temperature (Fig. 3 *D* and *E*), thermogenic gene expression (Fig. 3*F*), or UCP1 protein abundance (Fig. 3 *G* and *H*) in mice expressing hM4Di in the rPAG, whereas J60 had no effect in mCherry controls. These results identify the rPAG as a critical downstream relay mediating VMH^SF1^-driven thermogenic activation in brown adipose tissue.

### Chemogenetic Inhibition of PVT Neurons Abolishes VMH^SF1^→PVT-Induced Lipolysis.

**Fig. 4.**
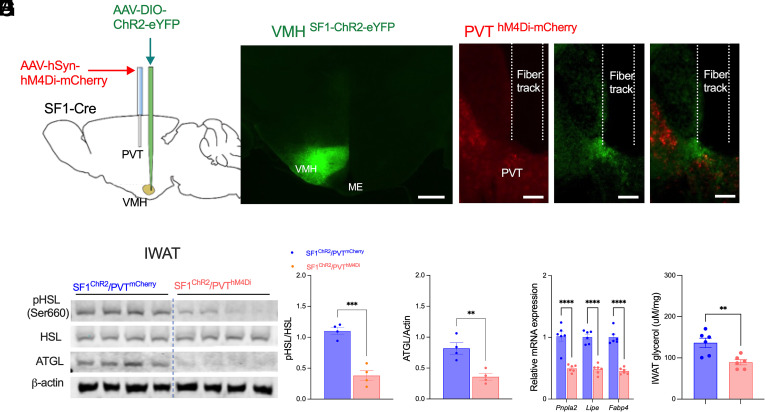
Chemogenetic inhibition of PVT neurons abolishes VMH^SF1^→PVT-driven lipolysis in white adipose tissue. (*A*) Schematic of viral delivery into the VMH (AAV-DIO-ChR2-eYFP) and PVT (AAV-hSyn-hM4Di or AAV-hSyn-mCherry), with optic fiber implantation above the PVT for projection-specific photostimulation. Mice received a single intraperitoneal injection of J60 (1 mg/kg) immediately before the 2-h photostimulation. (*B* and *C*) Representative fluorescence images showing ChR2 expression in VMH^SF1^ neurons (*B*) and terminal projections within the PVT along with hM4Di expression in PVT neurons (*C*). [Scale bar, 300 µm (*B*) and 100 µm (*C*).] (*D*–*F*) Representative western blots showing p-HSL, HSL, ATGL, and β-actin protein levels in iWAT from PVT mCherry- and ChR2- transduced mice (*D*). Quantification of fold changes in p-HSL relative to total HSL (*E*) and ATGL relative to β-actin (*F*). (*G*) qPCR analysis of lipolytic (*Pnpla2*, *Lipe*, and *Fabp4*) gene expression in iWAT. (*H*) Glycerol release in iWAT of PVT mCherry and hM4Di-transduced mice (n = 6 per group). Data are presented as mean ± SEM. Statistical analyses were performed using two-way ANOVA with Sidak’s post hoc test (*G*) or two-tailed Student’s *t* test (*E*, *F*, and *H*); each dot represents one animal. **P < 0.01, ****P < 0.0001.

To test whether PVT neuronal activity is necessary for VMH^SF1^→PVT-mediated lipolysis, AAV-hSyn-hM4Di or control AAV-hSyn-mCherry was injected into the PVT, and Cre-dependent AAV-hSyn-DIO-ChR2-eYFP was delivered to the VMH (Fig. 4 *A*–*C*). Administration of J60 (1 mg kg^−1^, i.p.) before photostimulation abolished stimulation-induced increases in p-HSL and ATGL protein levels (Fig. 4 *D*–*F*), lipolytic gene expression (Fig. 4*G*), or glycerol release (Fig. 4 *H* and *I*) in iWAT of hM4Di-expressing mice, but not in mCherry controls. These findings identify the PVT as an essential relay through which VMH^SF1^ neurons drive lipolytic activity in white adipose tissue.

### ChR2-Assisted Mapping Identifies a Monosynaptic VMH^SF1^ Output Pathway Capable of Driving Downstream Neuronal Firing.

**Fig. 5.**
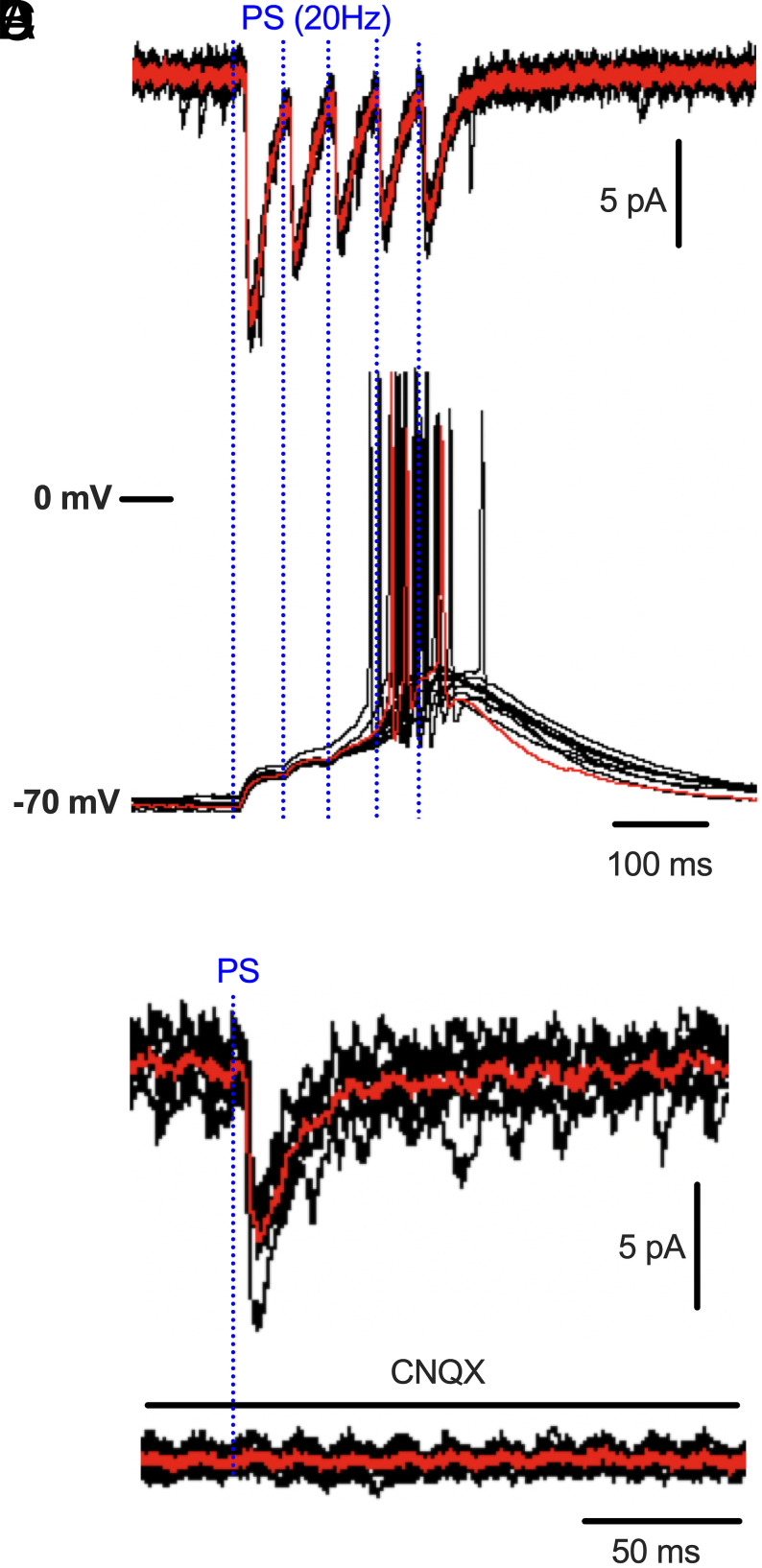
ChR2-assisted mapping reveals a monosynaptic, glutamatergic VMH^SF1^→PVT projection capable of driving PVT neuronal firing. (*A*) Photostimulation (20 Hz blue-light pulse trains) of VMH^SF1^ axon terminals in the PVT evokes robust, time-locked excitatory postsynaptic currents (EPSCs) in PVT neurons expressing ChR2-eYFP. Trace represents one of six neurons. (*B*) Under current-clamp configuration, the same stimulation paradigm reliably elicits action potentials in PVT neurons, indicating that VMH^SF1^ inputs provide sufficient excitatory drive to recruit postsynaptic firing. (*C*) Photostimulation (1 Hz)-evoked EPSCs persist in the presence of TTX (1 μM) and 4-AP (100 μM), consistent with monosynaptic terminal release. (*D*) EPSCs are completely abolished by the AMPA/kainate receptor antagonist CNQX (10 μM), confirming glutamatergic transmission.

To determine whether VMH^SF1^ neurons form monosynaptic connections with downstream targets, we performed ChR2-assisted circuit mapping in acute slices containing the PVT. Acute brain slices containing PVT were prepared from mice expressing ChR2-eYFP selectively in VMH^SF1^ neurons, and whole-cell recordings were obtained from PVT neurons. Photostimulation of VMH^SF1^ axon terminals in the PVT using 20 Hz trains blue-light pulses reliably evoked time-locked excitatory postsynaptic currents (EPSCs) in PVT neurons (Fig. 5*A*). Under current-clamp configuration, the same stimulation paradigm elicited robust action potentials (Fig. 5*B*), demonstrating that VMH^SF1^ inputs provide sufficient excitatory drive to recruit PVT neuronal firing. Photostimulation (1 Hz)-evoked EPSCs persisted in the presence of TTX with 4-AP (Fig. 5*C*) and were completely abolished by the AMPA/kainate receptor antagonist CNQX (Fig. 5*D*), consistent with a monosynaptic VMH^SF1^→PVT connection (7). Together, these findings establish that VMH^SF1^ neurons form a functional monosynaptic glutamatergic pathway capable of driving PVT neuronal output and forms the synaptic basis through which this hypothalamic–thalamic pathway regulates adipose lipolysis. In contrast, equivalent synaptic mapping in the rPAG was not feasible because the region’s highly condensed and tightly interwoven cytoarchitecture prevented stable whole-cell patch-clamp recordings.

### Local Denervation of iBAT Abolishes VMH^SF1^→rPAG-Induced Thermogenesis.

**Fig. 6.**
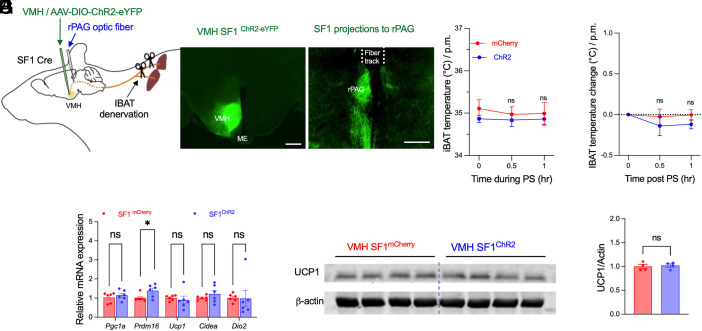
iBAT denervation abolishes VMH^SF1^→rPAG-driven thermogenesis in brown adipose tissue. (*A*) Schematic of viral delivery into the VMH (AAV-DIO-ChR2-eYFP or AAV-DIO-mCherry), with optic fiber implantation above the rPAG for projection-specific photostimulation. Nerves innervating iBAT were surgically transected. (*B* and *C*) Representative fluorescence images showing ChR2 expression in VMH^SF1^ neurons (*B*) and terminal projections within the rPAG (*C*). (Scale bar, 300 µm.) (*D* and *E*) iBAT temperature (*D*) and temperature change (*E*) before and during photostimulation (0, 0.5, 1 h) in mCherry- and ChR2-transduced mice (n = 5 per group). (*F*) qPCR analysis of thermogenic (*Pgc1α*, *Prdm16*, *Ucp1*, *Cidea*, and *Dio2*) gene expression in iBAT from mCherry and ChR2 mice. (*G* and *H*) Representative western blots showing UCP1 and β-actin protein levels in iBAT from mCherry- and ChR2- transduced mice (*G*). Quantification of fold changes in UCP1 relative to β-actin (*H*). Data are presented as mean ± SEM. Statistical analyses were performed using two-way ANOVA with Sidak’s post hoc test (*D*, *E*, and *F*) or two-tailed Student’s *t* test (*H*); each dot represents one animal. *P < 0.05, n.s. not significant.

To test whether VMH^SF1^→rPAG-driven thermogenesis requires intact local innervation, we surgically transected the nerves innervating iBAT following VMH^SF1^ neuron transduction with Cre-dependent AAV-ChR2-eYFP or control AAV-mCherry and optic fiber implantation above the rPAG (Fig. 6 *A*–*C*). In contrast to the robust thermogenic responses observed in intact mice (Fig. 1), iBAT denervation abolished VMH^SF1^→rPAG-induced thermogenesis, as indicated by unchanged iBAT temperature (Fig. 6 *D* and *E*), thermogenic gene expression (although *Prdm16* remained modestly elevated) (Fig. 6*F*), and UCP1 protein levels (Fig. 6 *G* and *H*). These data demonstrate that VMH^SF1^→rPAG-mediated thermogenic activation requires intact neural input to brown adipose tissue.

### Local Denervation of iWAT Abolishes VMH^SF1^→PVT-Induced Lipolysis.

**Fig. 7.**
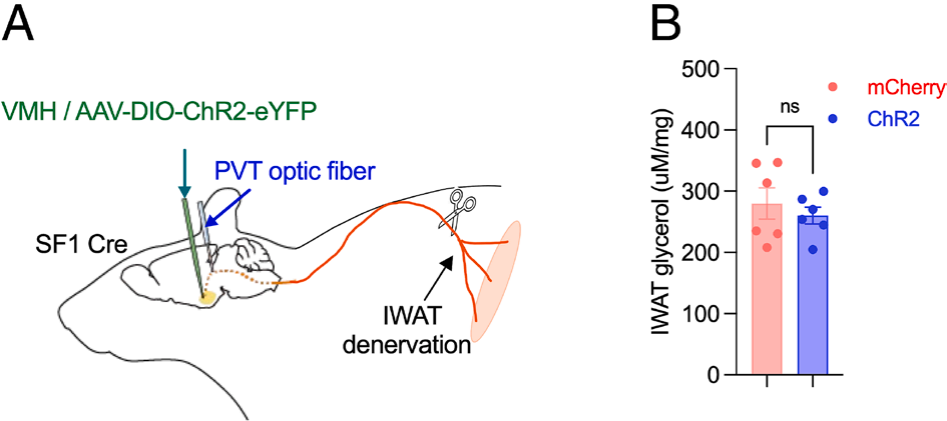
iWAT denervation abolishes VMH^SF1^→PVT-driven lipolysis in white adipose tissue. (*A*) Schematic of viral delivery into the VMH (AAV-DIO-ChR2-eYFP or AAV-DIO-mCherry), with optic fiber implantation above the PVT for projection-specific photostimulation. Nerves innervating iWAT were surgically transected. (*B*) iWAT glycerol release in mCherry- and ChR2-transduced mice (n = 6 per group). Data are presented as mean ± SEM. Statistical analyses were performed using two-tailed Student’s *t* test (*B*); n.s. not significant.

To determine whether VMH^SF1^→PVT-induced lipolysis depends on local innervation, we surgically transected the nerves innervating iWAT following VMH^SF1^ neuron transduction with Cre-dependent AAV-ChR2-eYFP or control AAV-mCherry and optic fiber implantation above the PVT (Fig. 7*A*). In contrast to the marked lipolytic responses in intact mice (Fig. 2), iWAT denervation abolished the VMH^SF1^→PVT-driven lipolysis, as photostimulation failed to increase glycerol release in ChR2-expressing mice relative to mCherry controls (Fig. 7*B*). This result indicates that VMH^SF1^→PVT-mediated lipolytic activation requires intact local innervation of white adipose tissue.

## Discussion

**Fig. 8.**
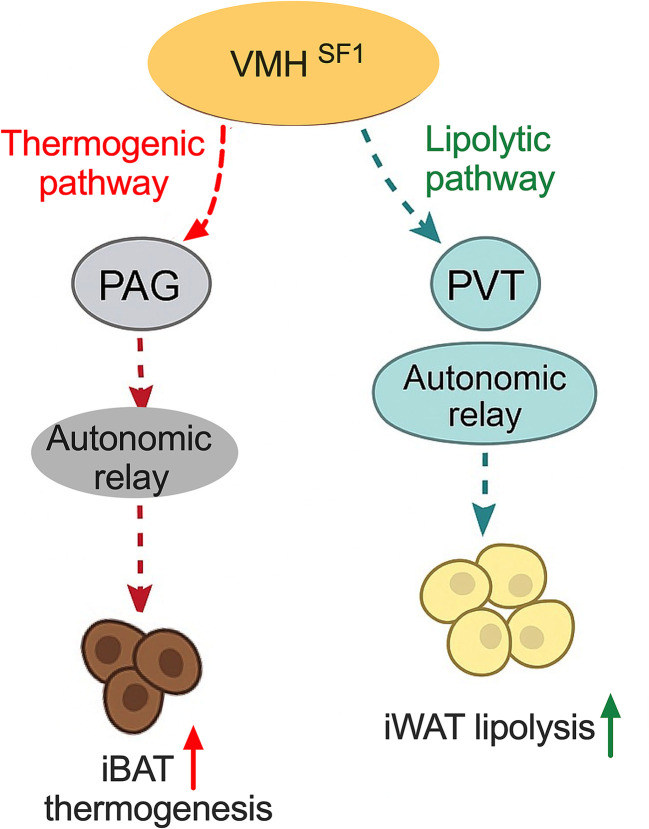
Projection-defined VMH^SF1^ output pathways regulate thermogenesis and lipolysis via target-specific autonomic relays. Schematic model illustrating two parallel VMH^SF1^ efferent pathways controlling adipose tissue metabolism. The VMH^SF1^→rPAG pathway (*Left*, red) functions as a thermogenic pathway that engages downstream autonomic relays to increase neural drive to interscapular brown adipose tissue (iBAT), thereby promoting heat production. In contrast, the VMH^SF1^→PVT pathway (*Right*, green) serves as a lipolytic pathway that engages distinct autonomic relays to increase neural drive to inguinal white adipose tissue (iWAT), thereby promoting lipid mobilization. Together, these projection-defined pathways partition thermogenic and lipolytic outputs, supporting a modular hypothalamic architecture for state-dependent regulation of peripheral energy metabolism.

Our study reveals two separate VMH^SF1^ projection pathways, distinguished both anatomically and functionally, exert different forms of control over adipose metabolism. As summarized in Fig. 8, signaling through the VMH^SF1^→rPAG pathway preferentially stimulates heat production in brown adipose tissue (iBAT), whereas the VMH^SF1^→PVT pathway preferentially facilitates lipid breakdown in white adipose tissue (iWAT). Although VMH^SF1^ neurons send collateral projections to more than one downstream region, the presence of branching axons does not eliminate the possibility of pathway-selective actions. Rather, our data support a model in which projection-defined outputs are recruited in a context-dependent manner, allowing VMH→rPAG and VMH→PVT pathways to differentially influence thermogenesis and lipid mobilization. Within this model, downstream networks engaged by the rPAG and PVT appear to activate different autonomic effector systems, thereby separating control of iBAT heat production from regulation of iWAT lipolysis. To determine the contributions of each projection independently, we selectively stimulated VMH^SF1^ axon terminals in either the rPAG or PVT. One physiological interpretation of these findings is that fasting or nutrient stress may shift VMH^SF1^ output toward the PVT, thereby mobilizing stored lipids without fully recruiting thermogenic programs, whereas acute cold exposure may preferentially increase the gain of VMH→rPAG pathways to rapidly increase sympathetic thermogenesis. Variation in synaptic strength, target-specific neuromodulatory tone, and the manner in which postsynaptic targets integrate incoming activity may further determine how VMH^SF1^ signaling is translated under different metabolic conditions. Together, these data provide direct support for the idea that the hypothalamus regulates peripheral energy metabolism through projection-specific mechanisms that separate thermogenic regulation from lipolytic processes.

Although SF1 neurons are broadly distributed throughout the VMH, our findings suggest that metabolic specificity is determined more by target-defined output pathways than by simple subregional localization within the VMH itself. This distinction underscores the importance of circuit-level approaches for parsing VMH function, as anatomical localization alone may not be sufficient to predict physiological consequence. In addition, whereas broad activation of VMH^SF1^ neurons is likely to engage several autonomic programs simultaneously, our projection-specific manipulations demonstrate that single output pathways are individually sufficient to drive distinct metabolic responses. This approach provides a critical foundation for subsequent studies aimed at testing whether these pathways are also required under conditions in which endogenous upstream signals are strongly engaged. VMH^SF1^ neurons innervate many downstream regions. We focused on the PVT and rPAG because these brain regions serve as functionally distinct integrative hubs and have recognized associations with lipid mobilization and sympathetic thermogenesis, respectively. The rPAG is well positioned anatomically to influence sympathetic circuits in the brainstem and spinal cord that participate in cold-defense responses and brown fat activation. By contrast, the PVT integrates hypothalamic metabolic information with arousal- and motivation-related processing and has also been linked to energy redistribution and white adipose lipolysis. Although other VMH projections may also participate in thermoregulatory control, our goal was not to catalog the full range of VMH efferent functions. Rather, we aimed to test whether VMH^SF1^ neurons can direct distinct peripheral metabolic programs through projection-defined engagement of distinct target regions.

Previous work established that global activation of the VMH increases sympathetic drive to iBAT, thereby promoting thermogenesis and energy expenditure (12, 31–33). Our results extend this framework by identifying the rPAG as a critical relay within this thermogenic pathway. The PAG projects to rostral medullary raphe neurons that regulate sympathetic outflow to brown adipose tissue (12, 21, 34–36). Consistent with this known circuit architecture, either chemogenetic inhibition of the rPAG or surgical denervation of iBAT prevented thermogenic activation by VMH^SF1^→rPAG stimulation, demonstrating that the rPAG acts as a key intermediate through which VMH^SF1^ neurons recruit sympathetic pathways to enhance brown fat thermogenesis. Importantly, the chemogenetic strategy used here suppressed local neuronal populations within the rPAG or PVT, rather than selectively silencing only those postsynaptic cells that directly receive VMH^SF1^ input. Accordingly, these experiments establish the requirement for activity within the target region itself, but they do not define the exact downstream neuronal subpopulation mediating the observed effect. We therefore cannot conclude whether VMH^SF1^ projections engage narrowly defined input-specific ensembles or instead recruit broader local circuit elements within these structures. Nevertheless, our data clearly show that adipose responses triggered by VMH^SF1^ activation depend on intact function of the relevant downstream target region. Because surgical denervation disrupts both sympathetic efferent fibers and sensory afferent inputs, which can themselves modulate adipose sympathetic tone, we interpret these experiments as showing that intact local innervation is necessary for VMH^SF1^-driven regulation of iBAT thermogenesis and iWAT lipolysis, rather than as evidence for selective elimination of sympathetic fibers alone.

In contrast to the rPAG pathway, stimulation of the VMH^SF1^→PVT pathway selectively increased triglyceride breakdown in iWAT without altering iBAT thermogenesis, indicating that this pathway primarily mediates lipid mobilization. Because the PVT integrates signals related to arousal, stress, and autonomic function (26–29, 37), this pathway may serve to couple lipid substrate release to behavioral or physiological states such as fasting, vigilance, or adaptation to stress. The functional divergence between the rPAG and PVT projections also reinforces the idea that VMH^SF1^ neurons are heterogeneous and likely composed of anatomically and molecularly distinct subpopulations (7, 38–41). Determining the molecular identities of PAG-projecting and PVT-projecting VMH^SF1^ neurons, and understanding how hormonal and nutrient signals differentially modulate these populations, should clarify how hypothalamic circuits coordinate internal and external cues to preserve systemic energy homeostasis. Although previous reports have shown that activation of VMH^SF1^ neurons can evoke defensive or arousal-like behaviors under certain stimulation conditions, we did not detect obvious freezing or burst-like locomotion activity using the stimulation parameters applied in this study. In addition, because the thermogenic and lipolytic responses depended on intact peripheral innervation of iBAT and iWAT, our findings support the interpretation that VMH^SF1^ control of adipose physiology is mediated mainly through projection-defined peripheral neural efferent mechanisms rather than as an indirect consequence of altered behavior.

An important direction for future work will be to determine whether these VMH^SF1^ output pathways are physiologically necessary under baseline conditions and during metabolic or thermal challenge using projection-specific inhibitory methods. For example, silencing the VMH^SF1^→rPAG and VMH^SF1^→PVT pathways chemogenetically, together with long-term metabolic phenotyping that include measurements of energy expenditure, food intake, movement, thermogenic competence, and body composition, should reveal whether these pathways participate in sustained control of adiposity and body weight. Such experiments should also clarify whether these projections are preferentially engaged during cold exposure, nutrient stress, or other physiological states, and whether their effects reflect immediate effector control or slower compensatory adaptations that emerge after prolonged circuit disruption.

Earlier studies identified AMPK signaling in VMH^SF1^ neurons as an important intracellular system for detecting energetic state and regulating energy expenditure (42). Our results extend that concept by showing that VMH^SF1^ neurons act through projection-defined output pathways that exert distinct metabolic functions. We propose that intracellular nutrient-sensing systems, including AMPK, act upstream of this circuit organization to modulate the recruitment and gain of specific projections, rather than producing a single uniform output across all VMH^SF1^ neurons. Under this model, metabolic and hormonal inputs converging on VMH^SF1^ neurons shape depot-specific sympathetic programs by preferentially engaging either rPAG-directed or PVT-directed pathways. This hierarchical arrangement offers a projection-level framework linking intracellular metabolic sensing to flexible and state-dependent regulation of adipose metabolism.

Collectively, as illustrated in Fig. 8, these findings define a modular VMH^SF1^ output architecture in which parallel rPAG- and PVT-directed projections independently regulate thermogenesis and lipolysis. This organizational principle suggests that discrete hypothalamic outputs can separate energy expenditure from substrate mobilization, thereby providing a neural basis for flexible metabolic control across physiological states. Beyond delineating two functionally distinct pathways, the present work also establishes a conceptual framework for projection-selective neuromodulatory strategies aimed at correcting specific metabolic impairments, such as reduced thermogenic capacity or defective lipid turnover, and may therefore point toward therapeutic avenues for obesity and related metabolic diseases.

## Materials and Methods

Experimental procedures were reviewed and approved by the Institutional Animal Care and Use Committees of Albert Einstein College of Medicine and were carried out in accordance with U.S. National Institutes of Health standards for the care and use of laboratory animals.

### Animals.

Male SF1-Cre mice (8 to12 wk old; strain #037533, The Jackson Laboratory) were used for all experiments. This mouse line has been previously validated and shown to exhibit selective Cre recombinase expression in steroidogenic factor-1 (SF1) neurons of the ventromedial hypothalamus. Animals were housed under controlled conditions with ambient temperature maintained at 22 to 25 °C and relative humidity at 40 to 60%, on a 12-h light/dark cycle with lights on at 08:00 a.m. with ad libitum access to water and standard laboratory chow (#5001, LabDiet). After stereotaxic viral injection and optical fiber implantation, animals were housed individually to prevent damage to the head-mounted implants throughout the experimental period.

### Brain Stereotaxic Viral Delivery and Optical Fiber Implantation.

Following previously documented procedures (43), anesthesia was induced with 3% isoflurane and maintained at 1 to 2% isoflurane during the surgical procedure. Mice were secured in a stereotaxic frame (David Kopf Instruments, Tujunga, CA) and placed on a heating pad to maintain body temperature throughout surgery. After exposing the skulls through a small incision, one or two burr holes were made above the intended injection sites using a precision drill (David Kopf Instruments). Pulled-glass micropipettes with a tip diameter of about 20 to 40 µm were inserted bilaterally or unilaterally into the brain as required by the experimental design. Viral infusions were controlled with a Narishige micromanipulator at a rate of 20 nL/min, and the pipette remained in place for 15 min after the final injection to assure adequate viral delivery. To target VMH neurons in *SF1*-Cre mice, either AAV-EF1α-DIO-hChR2-EYFP-WPRE-HGHpA (7 × 10^12^ vg/mL; Addgene) or AAV-hSyn-DIO-mCherry (control; 1.5 × 10^13^ vg/mL; Addgene) was unilaterally injected into the VMH (AP: –1.58 mm, ML: –0.3 mm, DV: –5.5 mm from bregma). Each VMH injection consisted of 200 nL delivered at 50 nL/min, and the pipette was kept in place for additional 5 min before withdrawal to reduce reflux along the injection tract. For manipulation of downstream regions, either AAV-hSyn-hM4Di-mCherry (7 × 10^12^ vg/mL; Addgene) or AAV-hSyn-mCherry (control; 5 × 10^12^ vg/mL; Addgene) was injected into the rPAG (AP: –2.45 mm, ML: –0.2 mm, DV: –3.0 mm from bregma) or the PVT (AP: –1.55 mm, ML: –0.2 mm, DV: –2.8 mm from bregma).

For optogenetic studies, a fiber-optic cannula (1.25 mm ceramic ferrule) (Thorlabs) was implanted above either the rPAG (AP: –2.45 mm, ML: –0.2 mm, DV: –2.5 mm from bregma) or the PVT (AP: –1.55 mm, ML: 0.0 mm, DV: –2.5 mm from bregma). In experiments targeting the rPAG, the fiber was implanted ipsilateral to the VMH injection site so that stimulation was restricted to virally transduced projections. For PVT experiments, the cannula was positioned at the midline. Cannulas were fixed to the skull using stainless-steel screws and dental cement. After surgery, mice received meloxicam (5 mg/kg, i.p.), recovered on a heated recovery pad, and were then returned to individual housing. Animals were monitored each day and allowed a minimum recovery period of 2 wk before the beginning of photostimulation experiments.

### Chemogenetic Inactivation of rPAG and PVT Neurons.

For inhibition studies, *SF1*-Cre mice previously injected in the rPAG or PVT with AAV-hSyn-hM4Di-mCherry, along with control mice expressing AAV-hSyn-mCherry, received an intraperitoneal administration of the DREADD agonist JHU37160 (J60; 1 mg/kg; Cayman Chemical). J60 was administered immediately before photostimulation so that ligand delivery and light stimulation began at the same time. This schedule was chosen to keep experimental conditions uniform across groups, reduce variability associated with handling immediately before stimulation, and ensure that neuronal inhibition was present during the stimulation period and subsequent measurements. Because the physiological readouts examined here, namely iBAT thermogenesis and iWAT lipolysis, emerge over a period of 10s of min, data analyses focused on sustained response intervals during which hM4Di- mediated suppression was expected to remain effective. Our prior work showed that J60 rapidly inhibited hM4Di-expressing neurons in vivo (7), which supports the timing used here. Under these conditions, chemogenetic suppression of either the PVT or rPAG significantly reduced the metabolic responses evoked by photostimulation, confirming that the inhibition strategy was effective.

### In Vivo Optogenetic Stimulation.

As stated in our prior studies (7, 43), 2 to 3 wk after surgery, fiber-optic cables with a 200 μm core diameter (BFH48-200-Multimode, NA 0.48; Thorlabs) were attached to the implanted cannulas by ceramic mating sleeves (Thorlabs). Mice were then placed individually in modified home cages that permitted free movement during the experiment. Before testing, animals were habituated to these recording cages for at least 1 wk, with ad libitum access to standard chow and water. On the test day, the optic fiber cable was connected to the cannula at 09:00 a.m. to allow acclimation before stimulation. Photostimulation (PS) was applied for 2 h from 16:00 to 18:00 using a 473 nm blue laser (Laserglow Technologies) controlled by a waveform generator (33220A, Agilent Technologies). The stimulation protocol consisted of 20 Hz pulses trains delivered for 1 s with 3 ms pulse width, repeated every 4 s for the full stimulation period. Light intensity at the fiber tip was adjusted to 15 to 20 mW before each experiment.

### iBAT and iWAT Denervation.

Following the detailed procedures in our recent study (43) with some modifications, surgical denervation of iBAT and iWAT was performed 1 wk after viral injection into the VMH of SF1-Cre mice. Mice were deeply anesthetized before the procedure. For iBAT denervation, a midline dorsal incision was made along the upper back to expose both iBAT pads. The medial–ventral aspect of each pad was gently separated to visualize the nerve bundles located beneath the tissue. Under a stereomicroscope, five nerve branches innervating each iBAT pad were identified, isolated, and bilaterally transected. For iWAT denervation, an incision was made in the inguinal region to expose the adipose pad and its associated nerves. The lateral cutaneous femoral nerve (LCFN) and anterior cutaneous femoral nerve (ACFN) were identified and locally crushed using fine forceps approximately 2 mm proximal to their points into the iWAT depot. After the procedure, antibacterial powder was applied to the exposed tissue, and the incisions were closed with surgical sutures. Animals were given 1 wk to recover under standard postoperative conditions before subsequent experiments. Denervation was performed to interrupt neural input to targeted depot. Because this operation can disrupt both efferent and afferent fibers, the results were interpreted as reflecting loss of local innervation rather than selective removal of a single neural component.

### iBAT Temperature Measurement.

One wk after viral injection and fiber implantation, SF1-Cre mice were implanted with a temperature transponder IPTT-300 temperature transponder (BMDS) beneath the iBAT pads. Briefly, the iBAT was exposed through a dorsal midline incision, and each pad was gently lifted to allow placement of the transponder underneath the tissue. The incision was then sutured, and mice were allowed to recover for at least 1 wk before experimentation. In animals assigned to the iBAT-denervated group, the nerves innervating iBAT were surgically transected first, and the temperature transponder was implanted immediately afterward. On the day of testing, the fiber-optic cable was connected to the cannula at 09:00 a.m. to allow acclimation. Photostimulation was applied for 2 h between 16:00 and 18:00, and iBAT temperature was measured at the indicated time points using a Bluetooth-enabled wireless reader system (DAS-8027 BLU, BMDS).

### Adipose Tissue Collection and Histological Verification.

At 2 to 3 wk after stereotaxic viral injection and fiber implantation, mice underwent 2 h of optogenetic stimulation, with or without concurrent chemogenetic manipulation, between 16:00 and 18:00. Following our recently published protocols (43, 44), immediately after the experimental, animals were deeply anesthetized and killed humanely, and the iBAT and iWAT were carefully dissected, rapidly frozen on dry ice, and stored at –80 °C until downstream analyses, including mRNA measurements, protein analysis, and glycerol release assays, unless noted otherwise. To verify viral targeting, brains were collected, fixed overnight in 4% paraformaldehyde at 4 °C, and then transferred to 30% sucrose from cryoprotection until the tissue sank. Coronal sections of 40 μm thickness were cut at –20 °C with a cryostat (CM1950; Leica Microsystems) and examined by a fluorescence microscope to assess the location and extent of viral expression.

### RNA Extraction and qPCR.

As detailed in our recent studies (43, 44), total RNA was isolated from approximately 50 mg of frozen iBAT or iWAT using TRIzol reagent (Thermo Fisher Scientific). Complementary DNA was generated from total RNA using the High-Capacity cDNA Reverse Transcription Kit (Applied Biosystems). qPCR was performed using the LightCycler 480 SYBR Green I Master Mix (Roche) on a QuantStudio 3 Real-Time PCR System (Applied Biosystems). Relative transcript levels were calculated by the 2^-ΔΔCt method using β-actin (*Actb*) as the reference gene.

The primer sequences used in this study were as follows:

**Table:** 

*Gene*	*Forward (5’→3’)*	*Reverse (5’→3’)*
*Pnpla2*	*CCAACACCAGCATCCAGT*	*CAGCGGCAGAGTATAGGG*
*Cidea*	*TGCTCTTCTGTATCGCCCAGT*	*GCCGTGTTAAGGAATCTGCTG*
*Dio2*	*AATTATGCCTCGGAGAAGACCG*	*GGCAGTTGCCTAGTGAAAGGT*
*Fabp4*	*AAGGTGAAGAGCATCATAACCCT*	*TCACGCCTTTCATAACACATTCC*
*Lipe*	*CGCCATAGACCCAGAGTT*	*TCCCGTAGGTCATAGGAGAT*
*Pgc1α*	*TATGGAGTGACATAGAGTGTGCT*	*CCACTTCAATCCACCCAGAAAG*
*Prdm16*	*CCACCAGCGAGGACTTCAC*	*GGAGGACTCTCGTAGCTCGAA*
*Ucp1*	*GTGAACCCGACAACTTCCGAA*	*TGAAACTCCGGCTGAGAAGAT*
*Actb*	*GCTGTCCCTGTATGCCTCT*	*GTCTTTACGGATGTCAACG*

### Western blotting.

Briefly, freshly extracted iWAT and iBAT samples were quickly snap-frozen in liquid nitrogen and stored at −80 °C until processing. Frozen iBAT and inguinal iWAT samples (~50 mg each) were homogenized in ice-cold lysis buffer containing protease and phosphatase inhibitor cocktails (Roche). Following our recently documented protocols (43, 44), homogenates were centrifuged at 15,000 × *g* for 15 min at 4 °C, and supernatants were collected for protein quantification using the Bradford assay. Equal amounts of protein (30 µg) were combined with 4× Laemmli sample buffer, boiled at 100 °C for 5 min, and separated by SDS–PAGE, and transferred to PVDF membranes. Membranes were blocked for 1 h at room temperature with Protein-Free Blocking Buffer (927-80001, LI-COR) and then incubated overnight at 4 °C with primary antibodies against ATGL (1:1,000; #2133, Cell Signaling Technology), pHSL (1:1,000; 45,804, Cell Signaling Technology), HSL (1:1,000; #4107, Cell Signaling Technology), UCP1 (1:1,000; MA5-31534, Invitrogen), and β-actin (1:10,000; MAB8929, R&D Systems). Following TBST washes (0.1% Tween-20 in TBS), membranes were incubated for 1 h at room temperature with IRDye-conjugated secondary antibodies (LI-COR). Protein bands were then detected with the Odyssey Classic Imaging System (LI-COR) after additional washes. Band intensity was quantified using ImageJ software, and values were normalized to either β-actin or total HSL as appropriate.

### Ex Vivo Glycerol Release Assay.

After the indicated treatments, freshly collected iBAT and iWAT samples (~10 mg each) were incubated overnight at 37 °C in 150 µL assay buffer in 1.5 mL microcentrifuge tubes within a humidified incubator. After incubation, supernatants were collected and stored at –80 °C until all groups were ready for analysis. Before measurement, samples were thawed on ice and diluted 1:20 in assay buffer. Glycerol standards were prepared according to the manufacturer’s protocol, and glycerol reagent A (LIP-6-NC, ZenBio) was freshly reconstituted before use. For each reaction, 100 µL of diluted sample or standard was combined with 100 µL glycerol reagent A in a 96-well plate. The plate was gently mixed by pipetting and incubated at room temperature for 15 min, and absorbance was measured at 540 nm using a microplate reader (model 800 TS, BioTek). Glycerol concentrations were determined from the standard curve and normalized to the starting tissue weight.

### Electrophysiology and Circuit Mapping.

As described in our recent study (7), acute coronal brain slices containing the PVT were prepared from SF1-Cre mice that had received viral transduction of ChR2 in VMH^SF1^ neurons. After isoflurane anesthesia and decapitation, brains were rapidly removed into in ice-cold oxygenated cutting solution (95% O^2^/5% CO^2^) containing (in mM): 110 choline chloride, 2.5 KCl, 1.25 NaH^2^PO^4^, 2 CaCl^2^, 7 MgSO^4^, 25 D-glucose, 3.1 Na-pyruvate, and 11.6 Na-L-ascorbate (pH 7.3). Coronal sections (260 μm) were cut on a vibratome (VT1200S; Leica Microsystems), incubated at 34 °C for 30 min, and then held at room temperature until recording. For recordings, slices were transferred to a submerged chamber and perfused continuously at 1 to 2 mL/min with oxygenated artificial cerebrospinal fluid (ACSF) containing (in mM): 119 NaCl, 25 NaHCO^3^, 11 D-glucose, 2.5 KCl, 1.25 MgCl^2^, 2 CaCl^2^, and 1.25 NaH^2^PO^4^ (pH 7.3). PVT neurons were visualized with an upright microscope (BX51WI; Olympus), and ChR2-eYFP-labeled fibers were identified by fluorescence. Whole-cell recordings were obtained using borosilicate glass electrodes filled with internal solution containing (in mM): 125 K-gluconate, 15 KCl, 10 HEPES, 8 NaCl, 4 Mg-ATP, 0.3 Na-GTP, 10 Na^2^-phosphocreatine, and 2 EGTA (pH 7.3). Neurons were recorded in either voltage clamp mode to assess synaptic currents or current clamp mode to measure action potential firing, with membrane potential held at –70 mV in both cases. Signals were acquired at 10 kHz using a MultiClamp 700B amplifier (Molecular Devices). Digitization and data analysis were performed with Clampfit 10.7 software (Molecular Devices). Optogenetically evoked postsynaptic currents were triggered by 473 nm blue-light stimulation (CrystaLaser) delivered to the slice surface through an optical fiber. Light pulses were 3 ms duration and were controlled by Clampfit 10.7 software in synchrony with electrophysiological data acquisition.

### Statistical Methods.

Animals were assigned randomly to control and experimental groups. After histological verification of viral targeting, mice showing off-target injections or incomplete expression were excluded from analyses. Only mice with correct viral targeting were retained in the final datasets. Across experiments, the average success rate for viral targeting was approximately 80%. Each experiment was independently repeated at least twice, and averaged values were used for statistical analyses. Before experiments began, mice were habituated for 3 to 7 d to intraperitoneal (i.p.) vehicle injection and related handling procedures. Comparisons between two groups were made using unpaired Student’s *t* tests. When more than two groups were compared, one-way ANOVA followed by Tukey’s post hoc test was applied. For analysis involving repeated measurements across time and treatment conditions, two-way repeated-measures ANOVA or mixed-effects ANOVA with within-subject and between-subject factors were applied when appropriate. All statistical analyses were performed using Prism 10.6.1 (GraphPad Software).

## Supplementary Material

Appendix 01 (PDF)

## Data Availability

All study data are included in the article and/or *SI Appendix*.
