# Supplementary material for: Projection-defined hypothalamic outputs differentially regulate thermogenesis and lipolysis
Source: Proc Natl Acad Sci U S A. 2026 May 27;123(22):e2535878123. doi: 10.1073/pnas.2535878123 (PMC13229187; doi:10.1073/pnas.2535878123)
Supplement: Supplementary file 1 — Appendix 01 (PDF) [file pnas.2535878123.sapp.pdf]

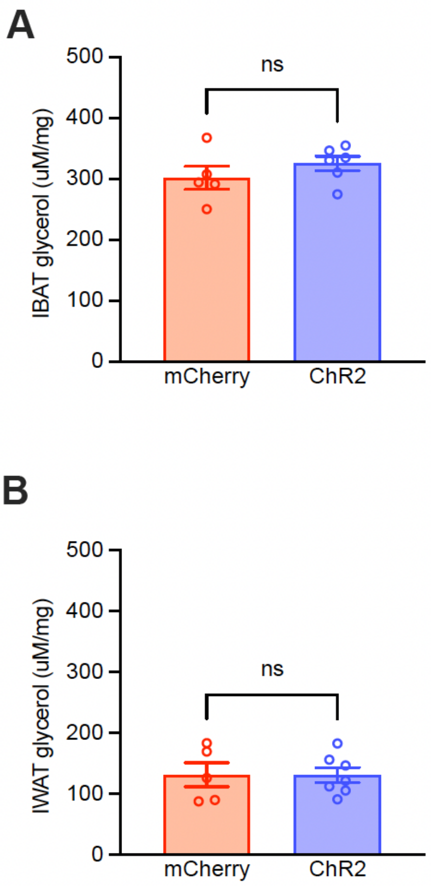


**Supplementary Figure 1 (related to Figure 1). Optogenetic activation of VMH$^{SF1}$→rPAG projections does not induce lipolytic responses in brown or white adipose tissue.**

(A) Glycerol release from iBAT in mCherry- and ChR2-transduced mice

(B) Glycerol release from iWAT in mCherry- and ChR2-transduced mice

Data are presented as mean ± SEM. Statistical analysis were performed using two-tailed Student's *t* test; each dot represents one animal. n.s. not significant.

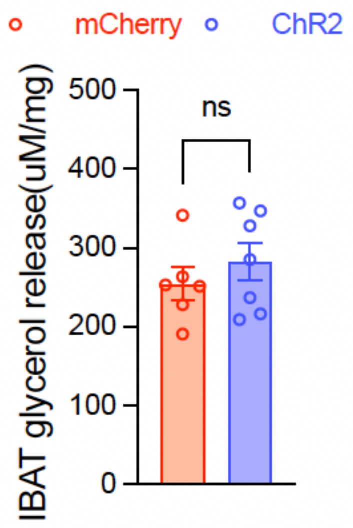


**Supplementary Figure 2 (related to Figure 2). Optogenetic activation of VMH$^{SF1}$→PVT projections does not induce lipolytic responses in brown adipose tissue.**

Glycerol release from iBAT in mCherry- and ChR2-transduced mice. Data are presented as mean ± SEM. Statistical analysis were performed using two-tailed Student's *t* test; each dot represents one animal. n.s. not significant.
